# Prevalence, Antimicrobial Susceptibility, Virulence and Genotyping of *Campylobacter jejuni* with a Special Reference to the Anti-Virulence Potential of Eugenol and Beta-Resorcylic Acid on Some Multi-Drug Resistant Isolates in Egypt

**DOI:** 10.3390/ani11010003

**Published:** 2020-12-22

**Authors:** Ahmed M. Ammar, El-Sayed Y. El-Naenaeey, Rania M. S. El-Malt, Attia A. El-Gedawy, Eman Khalifa, Shimaa S. Elnahriry, Marwa I. Abd El-Hamid

**Affiliations:** 1 Department of Microbiology, Faculty of Veterinary Medicine, Zagazig University, Zagazig 44519, Egypt; prof.ahmedammar_2000@yahoo.com (A.M.A.); sayedmyn@hotmail.com (E.-S.Y.E.-N.); mero_micro2006@yahoo.com (M.I.A.E.-H.); 2 Department of Microbiology, Animal Health Research Institute, Zagazig 44516, Egypt; 3 Tuberculosis Unit, Department of Bacteriology, Animal Health Research Institute, Giza 12618, Egypt; dr.attia31@yahoo.com; 4 Department of Microbiology, Faculty of Veterinary Medicine, Matrouh University, Matrouh 51511, Egypt; khalifa.eman@alexu.edu.eg; 5 Department of Bacteriology, Mycology and Immunology, Faculty of Veterinary Medicine, University of Sadat City, Menofia 32897, Egypt; kamelsamir95@yahoo.com

**Keywords:** *Campylobacter jejuni*, antimicrobial resistance, virulence, ERIC-PCR, eugenol, beta-resorcylic acid, invasion, cell line, virulence genes expression, broiler chickens

## Abstract

**Simple Summary:**

*Campylobacter jejuni* is the main reason for human foodborne bacterial enteritis globally. Contaminated chickens and their products are the principal reservoirs of *C. jejuni* and they are responsible for up to 80% of human campylobacter infection cases. In the current study, we determined the prevalence, antibiogram, the virulence factors encoding genes, and enterobacterial repetitive intergenic consensus-PCR (ERIC-PCR) profiles of *C. jejuni* isolated from chicken and human in Egypt, and we also assessed the effects of two phytochemicals (eugenol and beta-resorcylic acid) on the virulence of avian multi-drug resistant (MDR) and multi-virulent *C. jejuni* isolates. Our results revealed high prevalence of *C. jejuni* isolates (32.8%), and all isolates were MDR. Chicken and human *C. jejuni* isolates were clustered together as found in ERIC-PCR fingerprinting clusters II–V, which confirmed the genetic relatedness between both origins. Additionally, beta-resorcylic acid and eugenol reduced the invasion of MDR *C. jejuni* isolates to chicken intestinal epithelial cells and also minimized the transcription of *fla*A, *vir*B11, and *wla*N genes in the tested isolates. In conclusion, eugenol and beta-resorcylic acid could be used for minimizing the colonization and pathogenicity of *C. jejuni*; therefore, they could be utilized for controlling *C. jejuni* in broiler chickens and potentially for managing human campylobacteriosis.

**Abstract:**

*Campylobacter jejuni* is the leading cause of foodborne bacterial gastroenteritis in humans worldwide. Contaminated chickens and their products are the main sources of human campylobacteriosis. Therefore, this study aimed to detect the genotypic and virulence genes‘ profiles of multi-drug resistant (MDR) *C. jejuni* isolates and to assess the effects of sub-inhibitory concentrations (SICs) of eugenol and beta-resorcylic acid on the virulence of avian MDR *C. jejuni* isolates. These isolates were clustered together with the human isolates via enterobacterial repetitive intergenic consensus-PCR (ERIC-PCR) fingerprinting. A total of 345 samples were collected from human stool (100) and different chicken (245) samples in Sharkia Governorate, Egypt. Conventional phenotypic methods identified 113 isolates (32.8%) as *C. jejuni,* and all *C. jejuni* isolates were MDR and resistant to erythromycin and ampicillin. The genes *vir*B11, *wla*N, and *fla*A were detected in 52%, 36% and 100% strains, respectively. ERIC-PCR yielded 14 profiles and five main clusters. Interestingly, human and chicken *C. jejuni* isolates were clustered together in ERIC-PCR clusters II-V, which confirmed the genetic relatedness between the isolates from both origins. Beta-resorcylic acid and eugenol inhibited the invasion of *C. jejuni* isolates to chicken intestinal cells by 41.66–38.19% and 31.94–29.16%, respectively, and minimized the transcription of *fla*A, *vir*B11, and *wla*N genes in the tested isolates by real-time quantitative reverse transcription PCR (qRT-PCR). In essence, eugenol and beta-resorcylic acid are promising natural antimicrobials for minimizing the virulence of MDR *C. jejuni* in chickens, thereby managing human campylobacteriosis.

## 1. Introduction

For more than a century, knowledge about the public health significance of human campylobacteriosis has evolved. *Campylobacter jejuni* is a ubiquitous microorganism, and it is a commensal microorganism in the gastrointestinal tract of domestic animals and poultry. Human infection usually occurs through the consumption of contaminated water and food, mainly chicken and its products [1,2]. Campylobacter infection is a self-limiting disease and treatment with antimicrobials is not indicated in the majority of the cases; however, under specific clinical circumstances, antimicrobial therapy may be necessary. In these specific cases, the therapy may be complicated due to the emergence of multidrug resistant (MDR) campylobacter strains as a result of the wide uncontrolled usage of antimicrobials in agriculture and veterinary medicine [3]. 

For public health importance, it is essential to characterize the pathogenicity markers in *C. jejuni* isolates that are identified in food. *C. jejuni* has many putative virulence factors such as acid resistance, adhesions, cold and heat stress resistance [4], the flagella, its secreted proteins [5], capsule, hemolysin, and toxin production [6]. The Flagellin A (*fla*A) gene is a significant virulence marker in *C. jejuni* that defines the formation of flagella and thus, bacterial motility, adhesion, and invasion [7]. Moreover, *vir*B11, a plasmid encoded gene, is related to the invasion of the host cell [8], and the *wla*N gene is involved in the biosynthesis of β-1,3 galactosyltransferase production and lipooligosaccharide (LOS). Presumably, the later gene products mimic the ganglioside structure of the myelin sheath on nerve cells and lead to the emergence of Guillain-Barré syndrome (GBS), an acute peripheral polyneuropathy, after infection with *C. jejuni* [5]. 

*Campylobacter jejuni* genotyping is the final step in the characterization of strains to permit an efficient evaluation of their origins and/or relationship, which is essential to find the ideal solution for human and animal health problems [9]. Molecular typing depending on PCR-based methods such as enterobacterial repetitive intergenic consensus-PCR (ERIC-PCR) is evolved for *C. jejuni* genotyping [10]. ERIC-PCR is a simple, rapid and cheap technique in comparison with multilocus sequence typing (MLST), pulsed-field gel electrophoresis (PFGE) and whole-genome sequencing (WGS). Moreover, it has high discriminatory power and reproducibility, which favors its usage in *C. jejuni* typing to study the relationship of the isolates from various origins, the source attribution and the genetic diversity [11].

Chicken has been recognized as the main reservoir of *C. jejuni*, and it is expected to be the cause of up to 80% of campylobacteriosis in humans [5,12]. Human transmission commonly occurs by handling and ingestion of poultry meat and its products, which are contaminated throughout the slaughtering and carcass processing [12,13]. Thus, there is a significant need for effective strategies to control *C. jejuni* at the farm level by reducing the incidence of *C. jejuni* in chicken products, which leads to minimizing the product contamination and the incidence of human campylobacter infections [14]. Moreover, minimizing *C. jejuni* attachment and invasion of the intestinal epithelial cells and reducing their virulence genes production could potentially control the human campylobacter infection [15].

Several studies have focused on the ability of natural antimicrobial agents for controlling *C. jejuni* in poultry as a result of increasing the consumer’s preference for natural and safe products with minimal preservatives [16,17]. Herbal plant extracts have been commonly utilized as dietary supplements, food flavoring agents, and food preservatives to prevent food spoilage and to improve public health, since ancient times. Additionally, plant extracts have antimicrobial properties; therefore, they are utilized in herbal medicine for treating different illnesses [15]. Βeta-resorcylic acid (2,4-dihydroxybenzoic acid) is a phytophenolic complex, which is utilized as a flavor enhancer. It has important antimicrobial properties against major foodborne microorganisms such as *Listeria monocytogenes* [18]*, Salmonella* spp. [19] and *C. jejuni* [16]. Furthermore, it is classified by the FDA under “Everything Added to Food in the United States” [16,20]. Eugenol is another polyphenol complex, which is the main antimicrobial complex found in the clove oil (*Syzgium aromaticum*). The FDA classified these two phytochemicals as GRAS (Generally Recognized as Safe) with rapid biodegradation and minimal cytotoxicity, and they can be utilized in food as a good replacement for the antimicrobials [17,20,21].

Up to date, only Wagle and his collaborators have studied the effects of sub-inhibitory concentrations (SICs) of beta-resorcylic acid and eugenol on the invasion of human intestinal epithelial cells [22] and on the expressions of some virulence genes related to invasion and motility in *C. jejuni* isolates from the chicken origin in USA [16,17,22,23]. To complement their findings, we studied the efficacy of SICs of beta-resorcylic acid and eugenol on *C. jejuni* invasion of chicken intestinal epithelial cells and on the transcription levels of other virulence genes related to motility, adhesion, invasion and LOS production in MDR *C. jejuni* isolates from the chicken origin in Egypt; these isolates were genetically correlated with the human ones. 

Therefore, the aim of the current study was to (i) investigate the prevalence and antimicrobial resistance profiles of *C. jejuni* from chicken and human origins in Egypt, (ii) detect the virulence and genotypic profiles of multi-drug resistance (MDR) *C. jejuni* isolates and (iii) assess the efficacy of SICs of beta-resorcylic acid and eugenol on *C. jejuni* invasion of chicken intestinal epithelial cells and on the virulence genes’ expressions of *C. jejuni* isolates from the chicken origin via real-time quantitative reverse transcription PCR (qRT-PCR) assay; these isolates were genetically correlated with the human ones. 

## 2. Materials and Methods 

### 2.1. Sample Collection

A total of 345 various samples from human (100) and broiler chicken (245) sources were collected from various localities at Sharkia Governorate, Egypt during the period from September 2017 to March 2018. The chicken samples comprising cloacal swabs, neck skin, thigh meat, breast meat, cecal parts, liver, and gizzard (35 each) were collected from broiler chickens recently slaughtered in 15 different markets. Each sample represented a single bird. Furthermore, 100 human stool samples were collected from workers that were working in the chicken markets and suffering from diarrhea. The human stool samples were collected in private laboratories under nurse supervision. All participants gave their informed consent for inclusion before they participated in the study. The study was conducted in accordance with the Declaration of Helsinki and was approved by the research ethics committee of the Faculty of Veterinary Medicine, University of Sadat, Egypt (Approval No. VUSC-011-2-17). Twenty-five grams of each sample and the stool and cloacal swabs were collected in CampyloThioglycollate (Campy-Thio) broth base medium (Himedia, Mumbai, India) containing the Campylobacter selective supplement-I (Blaser-Wang, Himedia, Mumbai, India) [24]. The collected samples were aseptically transported in an icebox as soon as possible to the bacteriology laboratory for further examination. 

### 2.2. Isolation and Identification of Campylobacter jejuni

For *Campylobacter* spp. isolation, the collected samples in Campy-Thio broth were incubated for 24–48 h at 42 °C with less than 1 cm of headspace left in the culture vessels, which were kept with tightly-capped lids in darkness under microaerophilic conditions (85% N_2_, 10% CO_2_ and 5% O_2_) using CampyGen sachets (Oxoid, Cambridge, UK) and the anaerobic jar (Sigma-Aldrich, St. Louis, MI, USA). Following the enrichment step, 0.1 mL of the broth was inoculated onto the surface of modified charcoal cefoperazone deoxycholate agar (mCCDA) with CCDA selective supplement (Oxoid, Cambridge, UK) and the plates were incubated under microaerophilic conditions at 42 °C in darkness for 48 h. Additionally, three to four presumptive campylobacter colonies that had similar colonial morphology were further inoculated onto 5% sheep blood agar plates for 24–48 h at 42 °C under microaerophilic conditions in darkness. After incubation, suspected colonies were identified via Gram’s staining, motility test and biochemical identification using catalase, oxidase and rapid hippurate hydrolysis tests [25,26]. 

### 2.3. Antimicrobial Susceptibility Testing

Antimicrobial susceptibility tests were done using the standard disc diffusion method on Muller-Hinton agar (MHA) (Oxoid, Cambridge, UK). Few colonies of similar morphology were inserted into a tube containing 5 mL physiological saline (0.85%), and the turbidity of this suspension was adjusted to be equivalent to that of the 0.5 McFarland standard. The MHA plates were inoculated with the adjusted inoculum suspension and then they were incubated at 42 °C for 48 h under microaerophilic conditions. Ten antimicrobials (Oxoid, Cambridge, UK) belonging to seven different classes were used: norfloxacin (NOR, 10 µg), ciprofloxacin (CIP, 5 µg), nalidixic acid (NA, 30 µg), cephalothin (KF, 30 µg), erythromycin (E, 15 µg), kanamycin (K, 30 µg), gentamicin (CN, 10 µg), tetracycline (TE, 30 µg), ampicillin (AM, 10 µg) and trimethoprim/sulfamethoxazole (SXT, 25 µg). The degree of sensitivity of each isolate was determined by measuring the diameter of the inhibition zone around each disc and the results were interpreted according to the clinical and laboratory standards institute (CLSI) (Table 1) [27,28]. The MDR was defined as resistance to three or more unrelated antimicrobial agents. Finally, we determined the multiple antibiotic resistance (MAR) index for each isolate by utilizing the following formula: MAR = a/b, where (a) is the antimicrobials number to which the tested isolate was resistant and (b) represents the total number of antimicrobials used [29].

### 2.4. Molecular Grouping of MDR C. jejuni Isolates

All molecular investigations carried out in our study were conducted on the highly resistant *C. jejuni* isolates. 

#### 2.4.1. Extraction of DNA

Total DNA was extracted from the biochemically identified MDR *C. jejuni* isolates utilizing a Bacterial DNA Extraction Kit (QIAamp DNA Mini Kit; Qiagen, Valencia, CA, USA) according to the guidelines of the manufacture. 

#### 2.4.2. PCR Assays and Cycling Parameters

Uniplex PCR assays were performed for amplification of the *23S rRNA* gene of genus *Campylobacter, map*A gene of *C. jejuni* and three critical virulence genes (*wla*N, *fla*A, and *vir*B11). Moreover, ERIC-PCR was used to determine the genotypic profiles and the genetic association among the molecularly identified MDR *C. jejuni* isolates. All PCR reactions were done utilizing the Emerald Amp GT PCR master mix (Takara, Berkeley, CA, USA) according to the manufacturer’s guidelines. The specific genes and the primer sequences for all PCR assays are shown in Table 2. All amplification protocols were performed as previously described [30,31,32,33,34,35]. Gel electrophoresis and ethidium bromide staining (Sigma-Aldrich, St. Louis, MI, USA) were done as previously pronounced [36]. DNA of *C. jejuni* ATCC 33291 was utilized as a positive control and PCR grade water (no template DNA) was utilized as a negative control in uniplex PCR assays.

### 2.5. Assessment of the Efficacy of Phytochemicals on MDR C. jejuni Strains

Assessment of the Efficacy of beta-resorcylic acid and eugenol on the virulence of some MDR *C. jejuni* isolates as follow:

#### 2.5.1. Phytochemicals

Two phytochemicals were used to control *C. jejuni* virulence. Beta-resorcylic acid (Sigma-Aldrich, St. Louis, MO, USA) is a phytophenolic compound that is found in the angiosperms, and it is used as a food flavoring agent [22]. Additionally, eugenol (Sigma-Aldrich, St. Louis, USA) is another polyphenol compound that is the major antimicrobial component present in the oil of clove [15].

#### 2.5.2. Determination of Subinhibitory Concentrations of the Used Phytochemicals

The SICs of the used phytochemicals were determined against the selected MDR and multi-virulent avian *C. jejuni* strains that were clustered together with the human isolates via ERIC-PCR fingerprinting. 

Sterile 96-well microtitre plates containing twofold serial dilutions of beta-resorcylic acid and eugenol starting from 1000 μg/mL were made and separately inoculated with each *C. jejuni* strain (~5 × 10^5^ CFU/mL), and then the plates were incubated at 42 °C for 24 h under microaerophilic conditions. In the sterile 96-well microtitre plates, column 1 was designated for the positive control (containing bacterial inoculum but no phytochemicals); column 2 was designated for the negative control (containing phytochemicals but no bacterial inoculum); and columns 3–12 were designated for the test compounds. Results were expressed as averages of three independent experiments, and duplicate assays were performed for each experiment. Bacterial growth was determined by culturing from each well on mCCDA. The SICs of phytochemicals were determined as the highest concentration of the tested phytochemicals that did not inhibit *C. jejuni* growth after 24 h [22]. 

#### 2.5.3. Effects of Beta-Resorcylic Acid and Eugenol on Virulence of Avian MDR *C. jejuni* Isolates

The effects of eugenol and beta-resorcylic acid (at their SIC levels) on the virulence of some MDR and multi-virulent *C. jejuni* isolates from chicken origins were tested at both phenotypic and genotypic levels. The avian isolates were selected to represent the ERIC-PCR clusters, where they were clustered together with the human ones. 

##### Evaluation of Phytochemicals’ Effects on *C. jejuni* Invasion of Chicken Intestinal Epithelial Cells

The effects of beta-resorcylic acid and eugenol on *C. jejuni* virulence-associated phenotypes such as their potential for invasion of chicken embryo intestinal epithelial cells were determined as mentioned previously [15,22,37]. Briefly, monolayers of chicken embryo intestinal cells (Lohmann Tierzucht GmbH, Cuxhaven, Germany) were grown in 24-well tissue culture plates (Thermo Fisher, CA, USA) at ~10^5^ cells per well and inoculated with a mid-log culture (8 h) of each *C. jejuni* isolate (~10^6^ CFU/well) either alone without phytochemicals (control) or in combination with the determined SICs of the investigated phytochemicals. The inoculated monolayers were incubated at 42 °C for 1.5 h in a microaerophilic environment, and then they were washed twice in minimal broth media (Himedia, Mumbai, India) before being incubated for another 2 h in DMEM cell culture media containing gentamicin (100 μg/mL) (Invitrogen, Carlsbad, CA, USA) to kill the extracellular bacteria. Subsequently, the monolayer was washed with minimal broth media and lysed with 1 mL of 0.1% triton X-100 (Invitrogen, Carlsbad, CA, USA). The number of *C. jejuni* that invaded the epithelial cells was enumerated following the serial dilution in PBS and plating of the cell lysate on mCCDA plates (Oxoid, Cambridge, UK), and then incubation at 42 °C for 48 h in a microaerophilic environment. The inhibition of invasion was expressed as a percentage using the relative decrease in invasion by *C. jejuni* in the presence of phytochemicals. It was determined via the following formula: inhibition of invasion = 100(1 − T_B_/T_L_), where T_B_ and T_L_ are the numbers of invading *C. jejuni* cells (CFU/well) in the presence and absence of phytochemicals, respectively.

##### Evaluation of Phytochemicals’ Effects on *C. jejuni* Virulence Genes’ Expression by Real-Time Quantitative Reverse Transcription PCR Assay

The qRT-PCR assay was used to determine the efficacy of eugenol and beta-resorcylic acid SICs on *C. jejuni* virulence genes’ expression as mentioned previously [15,22]. Briefly, each selected *C. jejuni* strain was separately inoculated with the SICs of the tested phytochemicals in Campy-Thio broth to mid-log phase (8 h) at 42 °C under microaerophilic conditions. After incubation, aliquots of suspended cells were centrifuged and the supernatant was decanted and the pellet was used immediately for RNA extraction using QiampRNease Mini Kit (Qiagen, Valencia, CA, USA) following the guidelines of the manufacturer. Transcript levels of the investigated virulence genes of *C. jejuni* were determined in the presence or absence of eugenol and beta-resorcylic acid. Real-time PCR amplification was performed, in triplicates, in the Stratagene MX3005P real-time PCR machine (Thermo Fisher, CA, USA) using QuantiTect SYBR Green PCR Master Mix (Qiagen, Valencia, CA, USA) according to the guidelines of the manufacturer. A melting curve analysis was done to differentiate between the specific and non-specific amplification products. The *23S rRNA* gene was used as a normalizing agent (endogenous control). The relative expression levels of the investigated genes in *C. jejuni* cells exposed to the used phytochemicals compared to the non-exposed (control) cells were obtained by the delta-delta Ct (2^–**∆∆Ct**^**)** method [38], whereas fold changes (relative quantification, RQ) = 2^−ΔΔCt^, ΔΔCt = ΔCt treatment − ΔCt control, ΔCt = Ct tested gene − Ct endogenous control. If a ΔΔCt is equal to 0, the fold changes (RQ) will be 1, which indicates no change in gene expression between control and treatment. Moreover, RQ values below 1 indicate downregulation in the gene expression.

### 2.6. Statistical Analysis

ERIC-PCR genotypic patterns were converted to numeric bp by using the BioDocAnalyze (Biometra, Germany) program and then the ERIC-PCR data were converted to the binary code according to the absence or presence of each band. The Jaccard coefficient was used to determine the profiles’ similarity [39]. The dendrogram was achieved through the sequential hierarchical analysis and an unweighted pair group method with an arithmetic average (UPGMA). The dendrogram and the cluster analysis were achieved through the SPSS Inc. version 26 (IBM Corp., Armonk, NY, USA). The Fisher’s exact was used to study the differences in the prevalence of *C. jejuni*, antimicrobial resistance patterns and the prevalence of virulence genes from different sources. We also applied Pearson chi-square to identify the association between different typing methods. Moreover, we used independent samples *t*-test to compare the eugenol and beta-resorcylic acid effects on *C. jejuni* invasion and virulence genes’ expression. All tests were done using the SPSS Inc. version 26 (IBM Corp., Armonk, NY, USA). The *p* values of less than 0.05 were considered statistically significant. The antibiotyping, virulotyping and ERIC-PCR discriminatory powers (*D* values) were calculated using Simpson’s index of diversity [40]. The *D* value above 0.9 reflects good discrimination.

## 3. Results

### 3.1. Prevalence of C. jejuni in Different Samples at Sharkia Governorate, Egypt

According to the conventional identification results, a total of 113 campylobacter isolates were recovered from 345 different samples collected from various sources in Sharkia Governorate, Egypt (32.8%). All 113 isolates were identified phenotypically via conventional methods as *C. jejuni*. The prevalence of *C. jejuni* isolates in the collected samples is shown in Table 3. *Campylobacter jejuni* isolates were prevalent among chicken samples (33.9%), followed by human stool swabs (30%). Among chicken samples, *C. jejuni* isolates were highly distributed among cloacal swabs (54.3%) and cecal parts (40%), while they were isolated with lower incidence rates in the chicken gizzard (22.9%) and neck skin (25.7%) samples (Table 3). There was no significant difference in the prevalence of *C. jejuni* neither between human and combined chicken samples (*p* = 0.286) nor between different chicken samples (*p* = 0.118). Moreover, there was a significant difference in the prevalence of *C. jejuni* between human stool and chicken cloacal swabs samples (*p* = 0.014).

### 3.2. Antimicrobial Susceptibility Tests of C. jejuni Isolates

All 113 *C. jejuni* isolates were examined for their susceptibility to various antimicrobial agents of several groups as shown in Table 4. It has been found that all the examined isolates were resistant to erythromycin and ampicillin (100% each). Moreover, the majority of *C. jejuni* isolates were resistant to tetracycline (90.3%), trimethoprim/sulfamethoxazole (82.3%) and nalidixic acid (80.5%). On the other hand, the results revealed high frequencies of susceptibility to gentamycin (69.9%), followed by kanamycin (67.3%) and norfloxacin (59.3%) (Table 4). There were no statistically significant differences in the resistance profiles between *C. jejuni* isolates neither from combined chicken and human samples nor between different chicken samples in relation to the tested antimicrobials (*p* > 0.05). Furthermore, there was a statistically significant difference in the nalidixic acid resistance profile between *C. jejuni* isolates from chicken cloacal swabs and human stool samples (*p* = 0.016). Regarding the antimicrobial resistance patterns of *C. jejuni* isolates from different sources, it was observed that the resistance rates to tetracycline, nalidixic acid, and trimethoprim/sulfamethoxazole were more frequent among *C. jejuni* isolates from chicken (91.6, 83.1 and 79.5%, respectively) and human (86.7, 73.3 and 90%, respectively) origins (Table 4). Interestingly, it was found that all examined *C. jejuni* isolates were MDR. Moreover, 96.5% of the analyzed isolates were resistant to five or more antimicrobial agents and 25 *C. jejuni* isolates were resistant to 8 or 9 antimicrobials (22.1%) (Table 5).

Estimating the MAR indices for all *C. jejuni* isolates revealed that all the tested isolates had an index greater than 0.2, indicating a high-risk source of contamination, where the antimicrobials are often used. It was found that three *C. jejuni* isolates (2.7%) had an index of 0.9 (resistant to 9 antimicrobials); those were isolated from human stool swab (1) and chicken cecal part (1) and cloacal swab (1). Out of the 25 *C. jejuni* isolates that had MAR indices greater than 0.7 (resistant to 8 or 9 antimicrobials), 17 were isolated from the chicken origin (68%) and 8 from the human stool swabs (32%) (Table 5). There were no statistically significant differences in the MAR indices between *C. jejuni* isolates from combined chicken and human samples (*p* > 0.05). Furthermore, there was a statistically significant difference in the MAR index of 0.7 between *C. jejuni* isolates from chicken cloacal swabs and human stool samples (*p =* 0.002). Moreover, there was a statistically significant difference in the MAR index of 0.5 between the *C. jejuni* isolates from different chicken samples (*p* = 0.02). Herein, 24 antimicrobial resistance patterns were recorded among MDR *C. jejuni* isolates. Twenty-one *C. jejuni* isolates (18.6%) exhibited the most common resistant spectrum (AM, E, NA, CIP, TE, SXT) (Supplementary Table S1).

### 3.3. Molecular Identification of Genus Campylobacter and C. jejuni

Of the 113 isolates, 25 *C. jejuni* isolates that were resistant to 8 or 9 antimicrobials were submitted for PCR amplifications of *23S rRNA* and *map*A genes for molecular confirmation of genus *Campylobacter* and *C. jejuni*, respectively. These isolates were recovered from human (8) and broiler chicken (17) sources. All the 25 screened isolates (100%) were identified as genus *Campylobacter* and confirmed to be *C. jejuni*. 

### 3.4. Molecular Investigation of Virulence-Related Genes 

All molecularly confirmed *C. jejuni* isolates (25) were tested for the presence of three critical virulence genes that play important roles in *C. jejuni* pathogenesis (*fla*A, *vir*B11 and *wla*N). Of the 25 tested isolates, 13 (52%) were positive for the *vir*B11 gene, 9 (36%) were positive for the *wla*N gene and 25 (100%) were positive for the *fla*A gene. 

Regarding the distribution of the investigated virulence genes among the tested chicken and human *C. jejuni* isolates (25), it was found that *vir*B11 and *wla*N genes were more prevalent among avian *C. jejuni* isolates (52.9 and 41.2%, respectively), followed by the human ones (50 and 25%, respectively) (Supplementary Figure S1). Ten avian (58.8%) and four human (50%) *C. jejuni* isolates harbored at least two virulence genes. Moreover, six avian (41.2%) and two human (25%) *C. jejuni* isolates contained three investigated virulence genes (Supplementary Figure S2). 

Interestingly, four virulence gene profiles were presented among the examined *C. jejuni* isolates. Eleven MDR *C. jejuni* isolates (44%) exhibited the most common virulence gene profile (*fla*A) (Supplementary Table S2). There were no statistically significant differences in the virulence profiles of *C. jejuni* isolates neither from combined chicken and human samples nor from chicken cloacal swabs and human stool samples (*p >* 0.05).

### 3.5. Genotyping and Phylogenetic Characterization of MDR C. jejuni Isolates Using ERIC-PCR Technique

**Figure 1:**
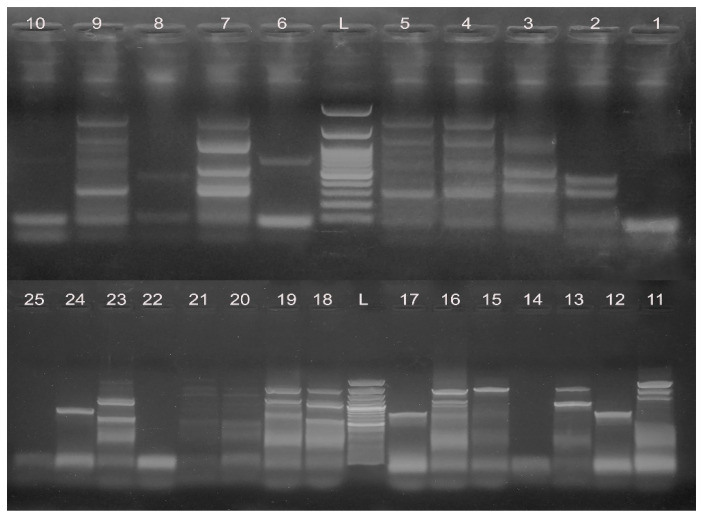
An agarose gel with ERIC-PCR products revealed by ethidium bromide staining. Lane L: 100 bp DNA ladder and lanes 1–25: ERIC-PCR fingerprints obtained for *C. jejuni* isolates from human and chicken samples with the following numbers: 1 H, 2 H, 3 H, 4 H, 106 Cg, 73 Ctm, 50 Ccp, 31 Ccs, 95 Cl, 96 Cl, 5 H, 107 Cg, 85 Cbm, 86 Cbm, 6 H, 74 Ctm, 75 Ctm, 7 H, 51 Ccp, 52 Ccp, 8 H, 32 Ccs, 33 Ccs, 64 Cns, and 65 Cns, respectively.

**Figure 2:**
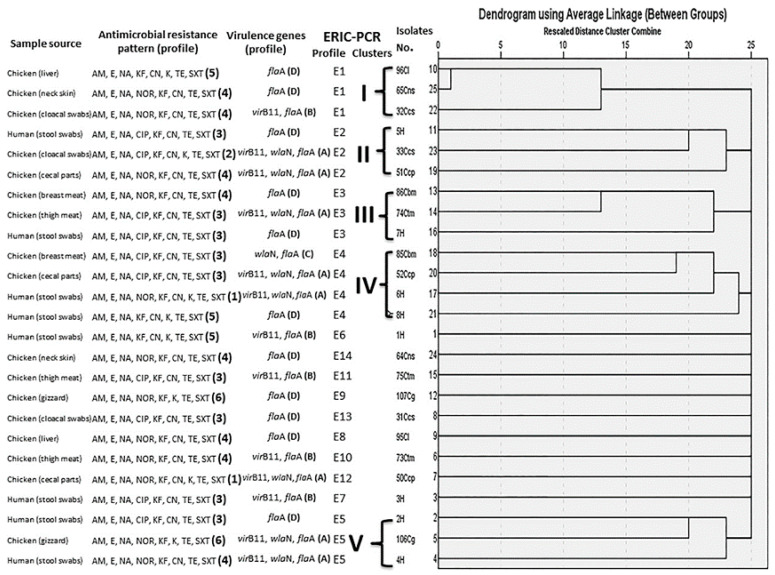
Dendrogram showing the relatedness of *C. jejuni* isolated from human and chicken origins as determined by ERIC-PCR fingerprinting correlating to the detailed phenotypic and genotypic criteria of these isolates. H: human, Ccs: chicken cloacal swabs, Ccp: chicken cecal parts, Cns: chicken neck skin, Ctm: chicken thigh meat, Cbm: chicken breast meat, Cl: chicken liver, Cg: chicken gizzard, AM: ampicillin, E: erythromycin, NA: nalidixic acid, CIP: ciprofloxacin, NOR: norfloxacin, KF: cephalothin, CN: gentamicin, K: kanamycin, TE: tetracycline, SXT: trimethoprim/sulfamethoxazole.

The typing and genomic relationships between the analyzed 25 MDR *C. jejuni* isolates were investigated by ERIC-PCR. This relationship can be distinguished based on the banding patterns generated with ERIC primers. ERIC PCR fingerprinting showed 14 profiles (E1 to E14) (Figure 1). Based on these profiles, a dendrogram was constructed to illustrate the relatedness of the examined 25 *C. jejuni* isolates from both avian and human sources, and it showed five main clusters for 16 isolates and nine separate isolates (Figure 2). Notably, clustering of human and chicken *C. jejuni* isolates together, as was found in clusters II–V, confirmed the genetic relatedness between the isolates from both origins (Figure 2). The Jaccard coefficient similarity matrix between poultry and human isolates of the same profile was 16.7–28.6% (18.2 and 20% in cluster II, 28.6% in cluster III, 20 and 25% in cluster IV and 16.7, and 22.2% in cluster V) (Supplementary Table S3).

### 3.6. The Discriminatory Power of Different Typing Methods for MDR C. jejuni Isolates

Six, 4 and 14 profiles of the 25 analyzed *C. jejuni* isolates were generated for antibiotyping, virulotyping, and ERIC-PCR methods (Figure 2). The discriminatory powers of the three methods used for typing of the 25 *C. jejuni* isolates were calculated based on their obtained profiles as summarized in Supplementary Table S4. Comparing the typing of *C. jejuni* isolates using these methods revealed that ERIC-PCR genotyping was the most effective method used for discrimination of the investigated isolates (*D* = 0.94) (Supplementary Table S4). Moreover, it was the highly discriminatory typing method for *C. jejuni* from both avian and human origins (*D* = 0.9559 and 0.9286, respectively). Of note, there was no correlation between antibiotyping, virulotyping, and ERIC-PCR fingerprinting (*p* = 0.871) in typing of *C. jejuni* isolates from chicken and human origins according to their obtained profiles. All phenotypic and genotypic criteria of the examined 25 *C. jejuni* isolates from different sources are presented in Figure 2.

Interestingly, six antimicrobial-resistant patterns were recorded among the examined 25 *C. jejuni* isolates. Nine of them (36%) exhibited the most common resistant profile (3; AM, E, NA, CIP, KF, CN, TE, SXT). Moreover, four virulence profiles were recorded among the tested isolates, and the most common profile (D: *fla*A) was present among 11 *C. jejuni* isolates (44%) (Figure 2). 

### 3.7. Effects of Beta-Resorcylic Acid and Eugenol on Virulence of Avian MDR C. jejuni Isolates

The effects of eugenol and beta-resorcylic acid at their SICs were tested on the virulence of four MDR and multi-virulent avian *C. jejuni* isolates (no. 106 Cg, 74 Ctm, 52 Ccp and 33 Ccs), which represented the four ERIC-PCR clusters (II–V), where they were clustered together with the human ones. The SIC levels of beta-resorcylic acid were 125 μg/mL for isolates no. 106 Cg, 52 Ccp and 33 Ccs and 62.5 μg/mL for isolate no. 74 Ctm; meanwhile, the SIC levels of eugenol were 125 μg/mL for isolates no. 74 Ctm and 52 Ccp and 62.5 μg/mL for isolates no. 106 Cg and 33 Ccs. All of the investigated isolates exhibited the virulence profile A (*vir*B11, *wla*N, *fla*A). Moreover, isolates no. 74 Ctm and 52 Ccp exhibited the most prevalent resistance profile (3; AM, E, NA, CIP, KF, CN, TE, SXT) and isolates no. 106 Cg and 33 Ccs showed the resistance profiles 6 (AM, E, NA, NOR, KF, K, TE, SXT) and 2 (AM, E, NA, CIP, KF, CN, E, TE, SXT), respectively.

#### 3.7.1. Efficacy of Sub-Inhibitory Concentrations of Beta-Resorcylic Acid and Eugenol on *C. jejuni* Invasion of Chicken Intestinal Cells

**Figure 3:**
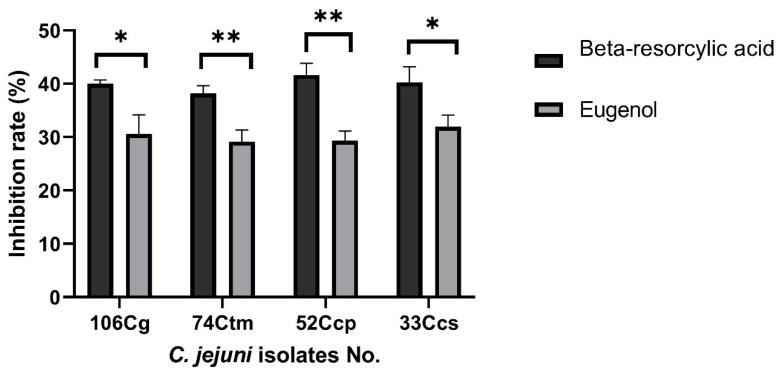
Inhibition of *C. jejuni* invasion of chicken intestinal epithelial cells under the impact of beta-resorcylic acid and eugenol. Values represent the inhibition percentage in comparison with the control untreated isolates, which were assigned a value of 0. Results were the average of three independent experiments, each containing duplicate samples (mean and SEM). * represents the significant differences between beta-resorcylic acid and eugenol, * *p* < 0.05, ** *p* < 0.01. Cg: chicken gizzard, Ctm: chicken thigh meat, Ccp: chicken cecal parts, Ccs: chicken cloacal swabs.

**Figure 4:**
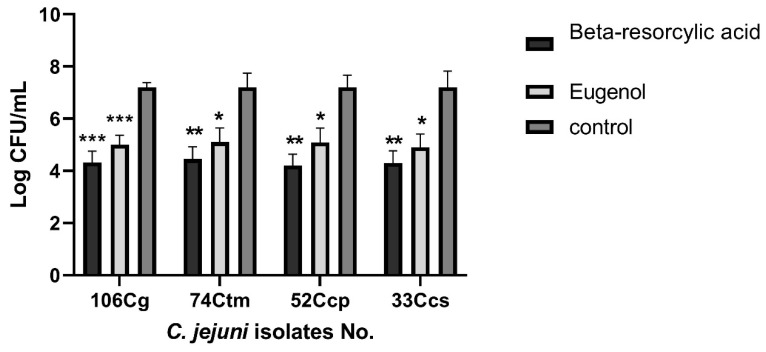
Effects of phytochemicals on *C. jejuni* invasion of chicken intestinal epithelial cells. Results were the average of three independent experiments; each contained duplicate samples (mean and SEM). * represents the significant differences between treatments and control, * *p* < 0.05, ** *p* < 0.01, *** *p* < 0.001. Cg: chicken gizzard, Ctm: chicken thigh meat, Ccp: chicken cecal parts, Ccs: chicken cloacal swabs.

Beta-resorcylic acid at SIC levels inhibited the invasion of *C. jejuni* isolates to chicken intestinal cells by 41.66–38.19% (Figure 3), where the number of invaded *C. jejuni* was 4.2–4.45 Log CFU/mL compared with 7.2 Log CFU/mL in control (no beta-resorcylic acid used), and *C. jejuni* invasion rate reduced by 3–2.75 Log CFU/mL (Figure 4).

Additionally, eugenol at SIC levels inhibited the invasion of *C. jejuni* isolates to chicken intestinal cells by 31.94–29.16% (Figure 3), where the count of invaded *C. jejuni* was 4.9–5.1 Log CFU/mL compared with 7.2 Log CFU/mL in control (no eugenol used) and *C. jejuni* invasion level reduced by 2.3–2.1 Log CFU/mL (Figure 4). These findings pointed to the role of beta-resorcylic acid and eugenol on minimizing *C. jejuni* invasion and colonization of chicken intestinal epithelial cells. 

There were statistically significant differences between the effects of eugenol and beta-resorcylic acid and the control (no phytochemicals used) on *C. jejuni* invasion of chicken embryo intestinal cells (*p* < 0.05). Furthermore, there were statistically significant differences between the effects of eugenol and beta-resorcylic acid on *C. jejuni* invasion of chicken embryo intestinal cells (*p* < 0.05). 

Interestingly, the lowest invasion level was detected in *C. jejuni* isolate no. 52 Ccp exposed to the SIC of beta-resorcylic acid (125 μg/mL), where the number of invaded *C. jejuni* was 4.2 log CFU/mL compared with 7.2 log CFU/mL in control (no beta-resorcylic acid used), and the invasion level was remarkably decreased by 3 log CFU/mL, and subsequently, the inhibition rate of *C. jejuni* invasion of chicken intestinal cells was 41.66%.

#### 3.7.2. Effects of Sub-Inhibitory Concentrations of Beta-Resorcylic Acid and Eugenol on the Expression of Critical Virulence Genes Using the qRT-PCR Assay

**Figure 5:**
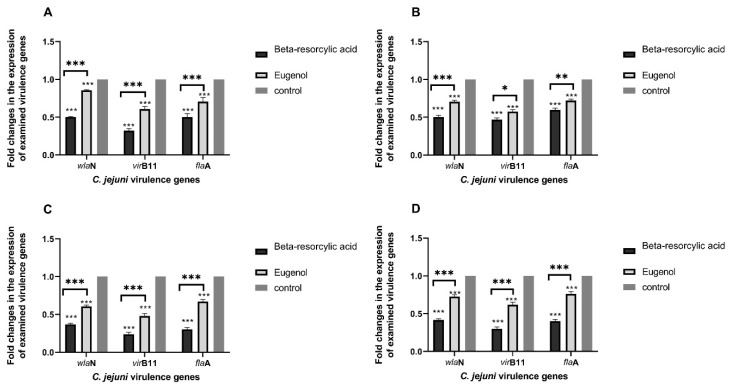
Fold changes in the expressions of *wla*N, *vir*B11 and *fla*A genes of four avian *C. jejuni* isolates; 106 Cg (**A**), 74 Ctm (**B**), 52 Ccp (**C**), and 33 Ccs (**D**) after treatment with SICs of eugenol and beta-resorcylic acid. Values represent the fold changes in comparison with the transcription levels of the control untreated isolates, which were assigned a value of 1. Results were the average of three independent experiments; each contained duplicate samples (mean and SEM). * represents the significant differences between treatments vs. control and between eugenol vs. beta-resorcylic acid, * *p* < 0.05, ** *p* < 0.01, *** *p* < 0.001. Cg: chicken gizzard, Ctm: chicken thigh meat, Ccp: chicken cecal parts, Ccs: chicken cloacal swabs.

Interestingly, in the current study, all tested genes (*fla*A, *vir*B11, and *wla*N) were found to be markedly down-regulated after exposure to SICs of the tested phytochemicals (Figure 5). It was interesting to observe that the lowest mRNA expression levels were ubiquitously detected in the isolates exposed to beta-resorcylic acid extract, where the transcriptional levels of tested genes were remarkably decreased (up to 0.2365-fold). These noticeable findings pointed to the wide range role of beta-resorcylic acid on the down-regulation of *C. jejuni* virulence genes. Likewise, the use of eugenol also repressed the expression levels of the examined genes (up to 0.4796-fold) (Figure 5). Both beta-resorcylic acid and eugenol suppressed the *vir*B11 gene to the maximum (up to 0.2365 and 0.4796-fold), followed by the suppression of *fla*A (up to 0.3015 and 0.6690-fold) and *wla*N (up to 0.3686 and 0.6071-fold) genes, respectively (Figure 5). There were statistically significant differences between the effects of eugenol and beta-resorcylic acid on the transcriptional modulation of *fla*A, *vir*B11 and *wla*N genes (*p <* 0.05).

## 4. Discussion

*Campylobacter jejuni* is a commensal microorganism in the gastrointestinal tract of domestic animals, especially poultry, which is considered the main source for human campylobacteriosis. In this study, we determined the high prevalence of *C. jejuni* in different samples collected from chicken and human origins in Sharkia Governorate, Egypt. The prevalence rate of *C. jejuni* observed in this study (32.8%) is partially similar to that obtained in a previous study carried out in United Kingdom (33.3%) [41], but it was higher than those reported in other studies carried out in Egypt (27.3%) [42], (26.9%) [43] and (20.3%) [44]. Herein, among chicken samples, *C. jejuni* isolates were highly distributed among cloacal swabs (54.3%), which is lower than that recorded in India (71%) [45] and Kenya (69%) [46]. Generally, the differences in *C. jejuni* prevalence between different sources among various studies may be attributed to the geographical location, climate factors, the type of examined samples, hygienic measures, the contamination status, health conditions, and the isolation and identification techniques of *C. jejuni* isolates [47].

Herein, all the examined isolates were resistant to ampicillin and erythromycin (100% each). These resistance rates were higher than those reported in previous studies conducted in South Korea (57.4 and 14.9%, respectively) [48] and Pakistan (15 and 10%, respectively) [49]. The differences in these resistance levels may reflect differences in hygiene and/or antibiotic use. In developing countries, antimicrobial resistance rates have markedly increased, and this could be attributed to the widespread and indiscriminate uses of antimicrobials in the veterinary and public health practices as the access to antimicrobials is very easy and they can be purchased without any prescription. The high incidence of erythromycinresistance detected in this work is alarming, because erythromycin is often the antibiotic of choice for treating human campylobacter infections, which are unresponsive to fluoroquinolones. This leads to a significant problem, where antimicrobial treatments become limited in numbers. Therefore, there is an urgent need for controlling the usage of erythromycin in human and animals.

In this work, all examined *C. jejuni* isolates were MDR and 96.5% were resistant to five or more antimicrobial agents. These results are in complete agreement with the results of a previous study in China (100 and 95.1%, respectively) [50]. Moreover, 22.1% of our *C. jejuni* were resistant to eight or more antimicrobials, which is higher than that reported in an earlier study in South Korea (5.4%) [48] and lower than that reported in a previous study in Egypt (100%) [51]. Furthermore, 2.7% of tested *C. jejuni* isolates had an MAR index of 0.9, which is partially similar to the result observed in a previous work in South Korea (3.2%) [48]. These results are alarming and were well related to the uncontrolled usage of drugs as growth promoters and prophylaxis in animal production [49,52,53].

Interestingly, the *vir*B11 gene was detected among 52% of the tested *C. jejuni* isolates. These findings were in contrast with the result of a previous study conducted in South Korea, which reported that the *vir*B11 gene was not detected in any of the tested isolates [54]. These variations in the incidence of this virulence gene may be due to the geographical location, sample types, and the isolates’ sources.

Herein, the *fla*A gene was detected in all tested *C. jejuni* isolates (100%). Our results are in complete agreement with a previous report in Egypt [55]. These observations suggest the fundamental role of the *fla*A gene as a virulence marker in *C. jejuni* strains, because *fla*A gene plays an important role in motility, adhesion and invasion of *C. jejuni* to the host intestinal epithelial cells.

In the current work, ERIC-PCR fingerprinting produced different profiles for chicken and human *C. jejuni* isolates. This is in agreement with a previous report in Zagazig, Egypt that detected various profiles for chicken and human *C. jejuni* isolates [56], confirming the genetic diversity of *C. jejuni.*

Notably, our human and chicken *C. jejuni* isolates were clustered together, as was found in ERIC-PCR clusters II–V. This is in complete agreement with earlier studies in Egypt [56] and India [57] that reported that poultry and human *C. jejuni* isolates were clustered together. These findings suggested that chicken has a significant role in human campylobacteriosis.

In this work, ERIC-PCR had higher discriminatory power than antibiotyping and virulotyping methods. This is in agreement with the result of an earlier scientific research in Zagazig, Egypt [56]. Moreover, there was no correlation between ERIC-PCR fingerprinting, antibiotyping and virulotyping, which is in complete agreement with the data observed in previous researches in Poland [58] and South Korea [54]. These results support the hypothesis that molecular typing depending on ERIC-PCR is better than the antibiotyping method.

Previous researches demonstrated that the SICs of different antimicrobial agents could change the invasion and gene expression of different virulence factors in bacteria, thus leading to minimizing their pathogenicity and virulence [15,16,59,60,61]. 

In the present study, beta-resorcylic acid at SIC values reduced *C. jejuni* invasion of chicken intestinal epithelial cells. This is similar to a previous study in the USA, which reported that beta-resorcylic acid minimized the invasion of avian *C. jejuni* isolates to human intestinal epithelial cells [22]. It was interesting to observe that eugenol at SIC values minimized the invasion of avian *C. jejuni* isolates to chicken intestinal epithelial cells. This is similar to a previous study in the USA, which revealed that eugenol reduced the invasion of human *C. jejuni* isolates to human intestinal epithelial cells [15].

Our results showed that eugenol and beta-resorcylic acid had similar effects on *C. jejuni* pathogenicity through the downregulation of critical virulence genes.

Interestingly, herein, *fla*A, *vir*B11, and *wla*N genes were found to be markedly down-regulated after exposure to SICs of beta-resorcylic acid. This is similar to previous studies in the USA, which reported that beta-resorcylic acid caused downregulation in the transcription levels of *C. jejuni* virulence genes coding for attachment (*cia*B and *jlp*A), motility (*fli*A, *mot*A, and *mot*B) and invasion (*cad*F) [16,22].

Moreover, *fla*A, *vir*B11, and *wla*N genes were found to be markedly down-regulated after exposure to SICs of eugenol. This is similar to previous studies in the USA, which revealed that eugenol caused downregulation in the transcription levels of *C. jejuni* virulence genes coding for motility (*fla*A) [17] and (*mot*A) [15] and invasion (*cad*F) [15]. Utilization of phytochemicals such as eugenol and beta-resorcylic acid, which are safe to be used in food, can be a safe and viable substitution to antimicrobials and chemical substances, which are used to control *C. jejuni* in broiler chicken products.

## 5. Conclusions

This study reported an alarming prevalence of MDR *C. jejuni* isolates from chicken and humans in Egypt. The *vir*B11 and *wla*N genes were more prevalent among the tested chicken *C. jejuni* isolates, followed by the human ones. The present research further supported the existence of genetic relatedness between examined human and chicken *C. jejuni* isolates. The SICs of eugenol and beta-resorcylic acid were found to minimize *C. jejuni* invasion to chicken intestinal epithelial cells and also markedly downregulated the expression of *vir*B11, *wla*N, and *fla*A genes in the examined MDR and multi-virulent avian *C. jejuni* isolates. Therefore, the current study recommends further studies to find the proper control approaches for MDR *C. jejuni* in Egypt and the potential utilization of beta-resorcylic acid and eugenol as food supplements for *C. jejuni* control in chickens.

## Figures and Tables

**Table 1:** Interpretation of inhibition zone sizes of different antimicrobial agents against *Campylobacter* species.

Antimicrobial Class	Antimicrobial Agent (Symbol)	Diameter of Inhibition Zone (mm) ^a^		
		S	I	R
Quinolones and fluoroquinolones	Nalidixic acid (NA)	≥19	14–18	≤13
	Ciprofloxacin (CIP)	≥24	21–23	≤20
	Norfloxacin (NOR)	≥17	13–16	≤12
β—lactams	Cephalothin (KF)	≥18	15–17	≤14
	Ampicillin (AM)	≥17	14–16	≤13
Aminoglycosides	Gentamicin (CN)	≥15	13–14	≤12
	Kanamycin (K)	≥18	14–17	≤13
Macrolides	Erythromycin (E)	≥16	13–15	≤12
Tetracyclines	Tetracycline (TE)	≥26	23–25	≤22
Sulfonamides	Trimethoprim/sulfamethoxazole (SXT)	≥16	11–15	≤10

^a^ Criteria for resistance according to zone diameter interpretive standards of infrequently isolated or fastidious bacteria defined by the CLSI [27] were employed as breakpoints for ciprofloxacin, erythromycin, and tetracycline. For other antimicrobials, CLSI [28] zone diameter interpretive standards for *Enterobacteriaceae* spp. were utilized, S: sensitive, I: intermediate sensitive, R: resistant.

**Table 2:** Oligonucleotide primer sequences and amplified PCR products of six target genes of *Campylobacter jejuni.*

Primer Name (Target Gene)	Primer Sequence (5’–3’)	Amplified Product (bp)	Reference
23 S (*23S rRNA*)	TATACCGGTAAGGAGTGCTGGAG	650	[31]
	ATCAATTAACCTTCGAGCACCG		
MapA (*map*A)	CTATTTTATTTTTGAGTGCTTGTG	589	[35]
	GCTTTATTTGCCATTTGTTTTATTA		
FlaA (*fla*A)	AATAAAAATGCTGATAAAACAGGTG	855	[33]
	TACCGAACCAATGTCTGCTCTGATT		
VirB (*vir*B11)	TCTTGTGAGTTGCCTTACCCCTTTT	494	
	CCTGCGTGTCCTGTGTTATTTACCC		
WlaN (*wla*N)	TTAAGAGCAAGATATGAAGGTG	672	[34]
	CCATTTGAATTGATATTTTTG		
ERIC	ATGTAAGCTCCTGGGGATTCAC	Variable	[30]
	AAG TAAGTGACTGGGGTGAGCG		

**Table 3:** Prevalence of *C. jejuni* isolates in different samples at Sharkia Governorate, Egypt.

Samples Source (No.)	Sample Type (Symbol, No)	No. of *C. jejuni* Isolates (%) *
Human (100)	Stool swabs (H, 100)	30 (30)
Broiler chicken (245)	Cloacal swabs (Ccs, 35)	19 (54.3)
	Cecal parts (Ccp, 35)	14 (40)
	Neck skin (Cns, 35)	9 (25.7)
	Thigh meat (Ctm, 35)	12 (34.3)
	Breast meat (Cbm, 35)	10 (28.6)
	Liver (Cl, 35)	11 (31.4)
	Gizzard (Cg, 35)	8 (22.9)
Total (345)		113 (32.8)

* The isolation rate was calculated concerning the total number of the examined samples from each source.

**Table 4:** Antimicrobial susceptibility patterns of 113 *C. jejuni* isolates from different sources.

AMA	No. of *C. jejuni* Isolates from Different Origins Showing Antimicrobial Susceptibility Patterns (%)								
	Human (30)			Chicken (83)			Total (113)		
	R	I	S	R	I	S	R	I	S
AM	30 (100)	-	0	83 (100)	-	0	113 (100)	-	0
E	30 (100)	-	0	83 (100)	-	0	113 (100)	-	0
NA	22 (73.3)	-	8 (26.7)	69 (83.1)	-	14 (16.9)	91 (80.5)	-	22 (19.5)
CIP	14 (46.7)	-	16 (53.3)	43 (51.8)	-	40 (48.2)	57 (50.4)	-	56 (49.6)
NOR	7 (23.3)	5 (16.7)	18 (60)	16 (19.3)	18 (21.7)	49 (59)	23 (20.4)	23 (20.4)	67 (59.3)
KF	23 (76.7)	0	7 (23.3)	62 (74.7)	6 (7.2)	15 (18.1)	85 (75.2)	6 (5.3)	22 (19.5)
CN	8 (26.7)	4 (13.3)	18 (60)	15 (18.1)	7 (8.4)	61 (73.5)	23 (20.4)	11 (9.7)	79 (69.9)
K	3 (10)	12 (40)	15 (50)	5 (6)	17 (20.5)	61 (73.5)	8 (7.1)	29 (25.7)	76 (67.3)
TE	26 (86.7)	1 (3.3)	3 (10)	76 (91.6)	1 (1.2)	6 (7.2)	102 (90.3)	2 (1.8)	9 (8)
SXT	27 (90)	-	3 (10)	66 (79.5)	-	17 (20.5)	93 (82.3)	-	20 (17.7)

AMA: antimicrobial agent, AM: ampicillin, E: erythromycin, NA: nalidixic acid, CIP: ciprofloxacin, NOR: norfloxacin, KF: cephalothin, CN: gentamicin, K: kanamycin, TE: tetracycline, SXT: trimethoprim/sulfamethoxazole, S: sensitive, I: intermediate sensitive, R: resistant.

**Table 5:** Multiple antibiotic resistance indices and resistance profiles of *C. jejuni* isolates from different sources.

MAR Index	No. of Antimicrobial Agents to Which *C. jejuni* Were Resistant	No. of Resistant *C. jejuni* Isolates from Different Origins (%)		
		Human (30)	Chicken (83)	Total (113)
0.3	3	0	1 (1.2)	1 (0.9)
0.4	4	0	3 (3.6)	3 (2.7)
0.5	5	7 (23.3)	17 (20.5)	24 (21.2)
0.6	6	15 (50)	35 (42.2)	50 (44.2)
0.7	7	0 (0)	10 (12)	10 (8.8)
0.8	8	7 (23.3)	15 (18.1)	22 (19.5)
0.9	9	1 (3.3)	2 (2.4)	3 (2.7)

MAR: multiple antibiotic resistance.

## Data Availability

Data is contained within the article or supplementary material.

## Supplementary Materials

The following are available online at https://www.mdpi.com/2076-2615/11/1/3/s1, Figure S1: Distribution of *Vir*B11, *wla*N and *fla*A genes among avian and human *C. jejuni* isolates, Figure S2: Distribution of one, two and three virulence genes among avian and human *C. jejuni* isolates, Table S1: Distribution of antimicrobial resistance patterns among multidrug-resistant *C. jejuni* isolates, Table S2: Distribution of virulence gene profiles among human and avian multi-drug resistant *C. jejuni* isolates, Table S3: Jaccard Coefficient similarity matrix between poultry and human *C. jejuni* isolates falling in ERIC-PCR clusters II–V, Table S4: Discriminatory power and profile numbers for different typing methods of 25 *C. jejuni* isolates.
