# Supplementary material for: Prevalence, Antimicrobial Susceptibility, Virulence and Genotyping of Campylobacter jejuni with a Special Reference to the Anti-Virulence Potential of Eugenol and Beta-Resorcylic Acid on Some Multi-Drug Resistant Isolates in Egypt
Source: Animals (Basel). 2020 Dec 22;11(1):3. doi: 10.3390/ani11010003 (PMC7822005; doi:10.3390/ani11010003)
Supplement: Supplementary file 1 [file animals-11-00003-s001.pdf]

Supplementary Material

Type of the Paper (Article)

Beta-Resorcylic Acid and Eugenol Minimized the Invasion and the Expression of Virulence Genes in Multi-Drug Resistant Avian *Campylobacter jejuni* isolates

Ahmed M. Ammar[1], El-Sayed Y. El-Naenaeey[1], Rania M. S. El-Malt[2*], Eman Khalifa[3], Shimaa S. Elnahriry[4], Attia A. El-Gedawy[5] and Marwa I. Abd El-Hamid[1]

[1] Department of Microbiology, Faculty of Veterinary Medicine, 44519, Zagazig University, Zagazig, Egypt; prof.ahmedammar_2000@yahoo.com (A.M.A.); sayedmyn@hotmail.com (S.Y.E.); mero_micro2006@yahoo.com (M.I.A)

[2] Department of Microbiology, Animal Health Research Institute, 44516, Zagazig, Egypt; raniaelmalt@yahoo.com

[3] Department of Microbiology, Faculty of Veterinary Medicine, Matrouh University, 51511, Matrouh, Egypt; khalifa.eman@alexu.edu.eg

[4] Department of Mycology and Immunology, Faculty of Veterinary Medicine, Sadat City University, 32897, Menofia, Egypt; kamelsamir95@yahoo.com

[5] Department of Bacteriology, Tuberculosis unit, Animal Health Research Institute, 12618, Dokki, Giza, Egypt; dr.attia31@yahoo.com

* Correspondence: raniaelmalt@yahoo.com

Table S1. Distribution of antimicrobial resistance patterns among multidrug-resistant *C. jejuni* isolates

Antimicrobial resistance pattern	No. of resistant *C. jejuni* isolates (%), source
**AM, E, SXT**	1 (0.9), Cg
**AM, E, KF, SXT**	3 (2.7), 2Ctm, 1Cbm
**AM, E, CIP, TE, SXT**	1 (0.9), Cg
**AM, E, NA, CIP, KF**	1 (0.9), Cg
**AM, E, NA, KF, TE**	2 (1.8), Cg
**AM, E, NA, TE, SXT**	2 (1.8), 1Cns, 1Cg
**AM, E, NA, CIP, TE**	2 (1.8), Cns
**AM, E, NOR, KF, SXT**	4 (3.5), H
**AM, E, KF, TE, SXT**	12 (10.6), 3H, 2Ccs, 2Ccp, 2Cns, 1Cbm, 2Cl
**AM, E, NA, CIP, KF, SXT**	2 (1.8), Cl
**AM, E, NA, NOR, TE, SXT**	1 (0.9), Cbm
**AM, E, NA, NOR, KF, TE**	1 (0.9), Cbm
**AM, E, NOR, KF, TE, SXT**	1 (0.9), H
**AM, E, NA, KF, TE, SXT**	10 (8.8), 4H, 1Ccp, 2Ctm, 1Cbm, 2Cl
**AM, E, NA, CIP, TE, SXT**	21 (18.6), 7H, 5Ccs, 3Ccp, 2Cns, 2Ctm, 2Cbm
**AM, E, NA CIP, KF, TE**	14 (12.4), 3H, 3Ccs, 2Ccp, 2Ctm, 1Cbm, 3Cl
**AM, E, NA, NOR, KF, TE, SXT**	4 (3.5), 3Ccs, 1Ctm
**AM, E, NA, CIP, KF, TE, SXT**	6 (5.3), 3Ccs, 3Ccp
**AM, E, NA, NOR, KF, K, TE, SXT**	2 (1.8), Cg
**AM, E, NA, KF, CN, K, TE, SXT**	3 (2.7), 2H, 1Cl
**AM, E, NA, CIP, KF, CN, TE, SXT**	9 (8), 4H, 1Ccs, 1Ccp, 2Ctm, 1Cbm
**AM, E, NA, NOR, KF, CN, TE, SXT**	8 (7.1), 1H, 1Ccs, 1Ccp, 2Cns, 1Ctm, 1Cbm, 1Cl
**AM, E, NA, NOR, KF, CN, K, TE, SXT**	2 (1.8), 1H, 1Ccp
**AM, E, NA, CIP, KF, CN, K, TE, SXT**	1 (0.9), Ccs
**Total**	113 (100)

H: human, Ccs: chicken cloacal swab, Ccp: chicken cecal part, Cns: chicken neck skin, Ctm: chicken thigh meat, Cbm: chicken breast meat, Cl: chicken liver, Cg: chicken gizzard, AM: ampicillin, E: erythromycin, NA: nalidixic acid, CIP: ciprofloxacin, NOR: norfloxacin, KF: cephalothin, CN: gentamicin, K: kanamycin, TE: tetracycline, SXT: trimethoprim/sulfamethoxazole.

Table S2. Distribution of virulence gene profiles among human and avian multi-drug resistant *C. jejuni* isolates

Virulence gene profile	No of *C. jejuni* isolates from different origins (%) harboring virulence gene profile		
	**Human (8)**	**Chicken (17)**	**Total (25)**
*vir*B11, *wla*N, *fla*A	2 (25%)	6 (35.3%)	8 (32%)
*vir*B11, *fla*A	2 (25%)	3 (17.6%)	5 (20%)
*wla*N, *fla*A	-	1 (5.9%)	1 (4%)
*fla*A	4 (50%)	7 (41.2%)	11 (44%)

Table S3. Jaccard Coefficient similarity matrix between poultry and human *C. jejuni* isolates falling in ERIC-PCR clusters II-V

ERIC-PCR clusters (*C. jejuni* isolate No)	II (33Ccs)	II (51Ccp)	III (74Ctm)	III (86Cbm)	IV (85Cbm)	IV (52Ccp)	V (106Cg)
II (5H)	**20%**	**18.2%**	0	0	0	0	0
III (7H)	0	0	**28.6%**	0	0	0	0
III (86Cbm)	0	0	50%	100%	0	0	0
IV (6H)	0	0	0	0	0	**25%**	0
IV (8H)	0	0	0	0	0	**20%**	0
IV (85Cbm)	0	0	0	0	100%	25%	0
V (2H)	0	0	0	0	0	0	**22.2%**
V (4H)	0	0	0	0	0	0	**16.7%**

H: human, Ccs: chicken cloacal swabs, Ccp: chicken cecal parts, Ctm: chicken thigh meat, Cbm: chicken breast meat, Cg: chicken gizzard.

Table S4. Discriminatory power and profile numbers for different typing methods of 25 *C. jejuni* isolates

Typing technique	Source of isolates (No)	Number of profiles	Discriminatory index (*D*)
**Antibiotyping**	**Chickens (17)**	6	0.7647
	**Human (8)**	4	0.75
	**Total (25)**	6*	0.77
**Virulotyping**	**Chickens (17)**	4	0.7132
	**Human (8)**	3	0.7143
	**Total (25)**	4*	0.69
**ERIC-PCR**	**Chickens (17)**	12	0.9559
	**Human (8)**	6	0.9286
	**Total (25)**	14*	0.94

*Some profiles of human origin are included within those of the chicken origin

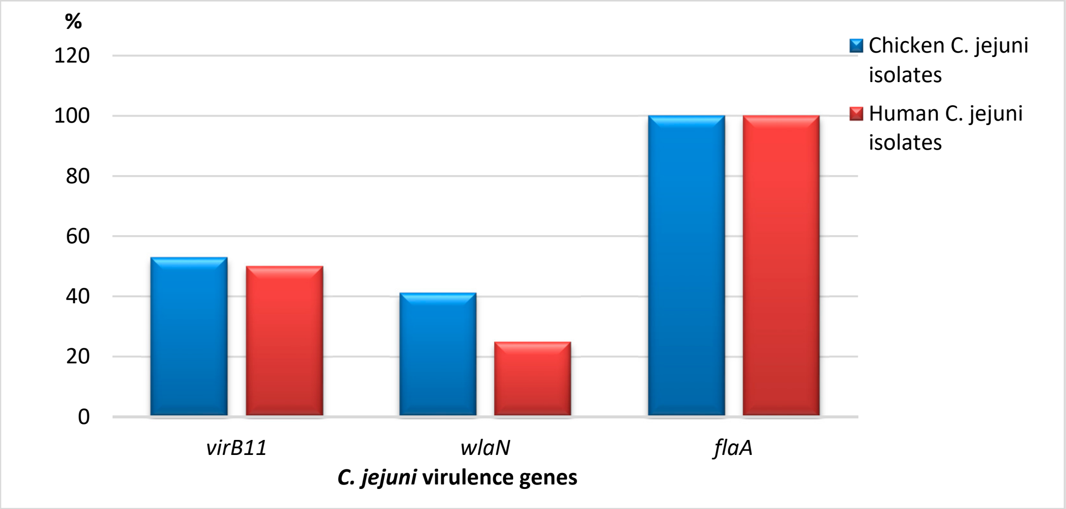

Figure S1. Distribution of *Vir*B11, *wla*N and *fla*A genes among avian and human *C. jejuni* isolates

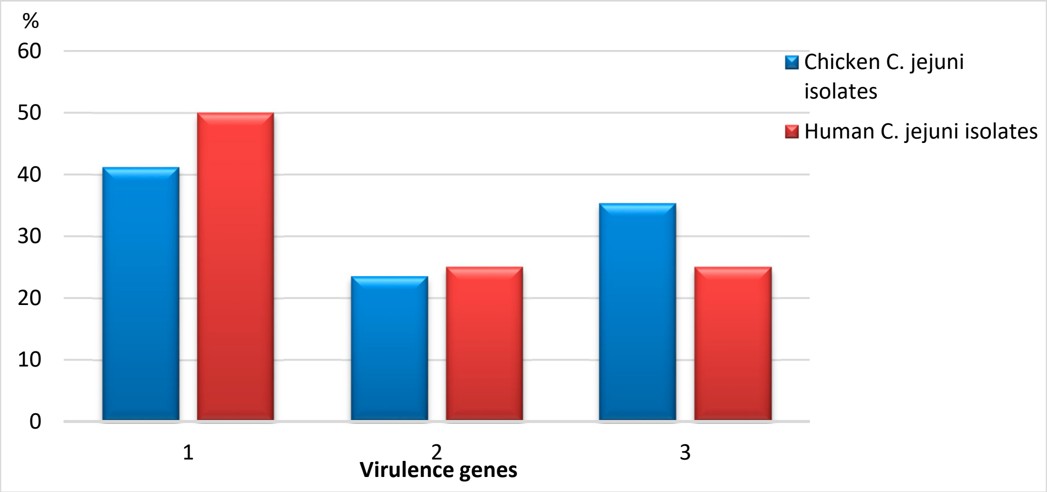

Figure S2. Distribution of one, 2 and 3 virulence genes among avian and human *C. jejuni* isolates
